# Exploring Caregivers’ Perspectives on Dementia Diagnostic Pathways and the Implementation of Artificial Intelligence (AI)‐Based Screening: A Qualitative Study

**DOI:** 10.1002/alz70858_099304

**Published:** 2025-12-25

**Authors:** Labhpreet Kaur, Daniel J. Blackburn, Alys Griffiths, Judith Rose Harrison

**Affiliations:** ^1^ University of Sheffield, Sheffield, South Yorkshire, United Kingdom; ^2^ Translational and Clinical Research Institute, Faculty of Medical Sciences, Newcastle University, Newcastle upon Tyne, England, United Kingdom

## Abstract

**Background:**

The current dementia diagnostic pathway uses pen‐and‐paper cognitive assessment tools. These findings are corroborated by family members and caregivers, who are asked to complete an informant report, a questionnaire or test used to identify changes observed in the person they care for and provide vital collateral information to support diagnoses. Recent research has begun exploring the use of digital and artificial intelligence (AI)‐based tools, such as CognoSpeak™, to automatically detect those with early signs of cognitive impairment and dementia. This study seeks to understand caregivers' views and experiences using the memory assessment pathway, the use of traditional cognitive‐ and informant‐based tools, and their perspectives on the potential use of AI‐based tools in supporting dementia diagnoses.

**Method:**

13 caregivers of people with dementia were recruited to take part in a semi‐structured interview, followed by a think‐aloud interview. The first semi‐structured interview explored caregivers’ views and experiences on (1) the memory assessment pathway, (2) current cognitive‐ and informant‐based tests and (3) their overall involvement in the diagnostic process. Caregivers were then asked to participate in a second think‐aloud interview, where they provided opinions on a pen‐and‐paper informant report, the Cambridge Behavioural Inventory (CBI‐R) and two automated assessment tools: CognoSpeak™ and MemoryChat. Interviews were analysed using thematic analysis to identify key themes.

**Result:**

Early findings highlight caregivers views and experiences using the memory assessment pathway, emphasising (1) a lack of education surrounding dementia and its symptoms, (2) describing the challenges in obtaining a timely diagnosis due to late or incorrect referrals, (3) concerns surrounding the use of standardised ‘box‐ticking’ tests in clinical assessments and (4) the importance of including and acknowledging informant reports to capture the extent of patient symptoms and providing a collateral history. Early findings also suggest mixed attitudes towards AI‐based tools, with caregivers appreciating their potential for early detection, remote access, ease and limited use of ‘rigid, box‐ticking’ questionnaires, but expressing concerns about reliability and technological unfamiliarity.

**Conclusion:**

Caregivers emphasised the need for timely and accurate dementia diagnoses, highlighting gaps in current pathways and tools. While AI‐based tools show promise, they must address caregiver concerns about reliability and accessibility.

